# Dyslexia as a Neurodevelopmental Disorder and What Makes It Different from a Chess Disorder

**DOI:** 10.3390/brainsci8100189

**Published:** 2018-10-19

**Authors:** Gorka Fraga González, Iliana I. Karipidis, Jurgen Tijms

**Affiliations:** 1 Department of Developmental Psychology, University of Amsterdam, Postbus 15916 1001 NK Amsterdam, The Netherlands; jurgentijms@gmail.com; 2 Rudolf Berlin Center, 44401 Amsterdam, The Netherlands; 3 Department of Child and Adolescent Psychiatry and Psychotherapy, Psychiatric Hospital, University of Zurich, 8032 Zurich, Switzerland; iliana.karipidis@kjpd.uzh.ch; 4 Neuroscience Center Zurich, University of Zurich and ETH Zurich, 8057 Zurich, Switzerland; 5 RID, 1018 WS Amsterdam, The Netherlands

**Keywords:** dyslexia, disorder, neurodevelopmental

## Abstract

The convenience of referring to dyslexia as a neurodevelopmental disorder has been repeatedly brought into question. In this opinion article, we argue in favor of the current diagnosis of dyslexia based on the criteria of harm and dysfunction. We discuss the favorable clinical and educational outcomes of a neuroscience-informed approach of dyslexia as a disorder. Furthermore, we discuss insights derived from neuroimaging studies and their importance to address problems related to developmental dyslexia.

## 1. Introduction

Individuals with dyslexia are characterized by specific and persistent reading problems. Prevalence rates vary widely, although usually it is estimated that 3–7% of the population suffers from dyslexia [1,2]. 

Over the years, the convenience of labeling dyslexia as a (neurodevelopmental) disorder has been repeatedly brought into question [3,4,5]. One of the main arguments against the term is that people differ in their reading proficiency along a continuum, and dyslexia is simply the lower-end tail of this continuum, cut-off from the rest of the distribution by some artificial, arbitrary criterion. To be a disorder, the problem in question should be of a categorical nature [4,6,7]. A logical consequence of this is that there is no need to categorize poor readers as a different group than good readers, some children simply are better at learning how to read than others. In a similar line of reasoning, the observed brain differences between dyslexics and non-dyslexics merely account for individual differences in reading skills, which will necessarily have a brain basis as any other cognitive ability has [4]. In sum, this view of dyslexia seems to emphasize individual differences in learning and performance, in contrast to the notion of typical/atypical development that is implied in terms such as ‘neurodevelopmental disorder’. 

Additional arguments against the use of the term dyslexia center on the alleged causes of poor reading. It has been claimed that what is called dyslexia is in fact the consequence of poor teaching methods [5] and that good-quality teaching would prevent children from becoming (labelled as) dyslexic. The argument, which in our opinion conflicts with the presence of dyslexia across very diverse educational systems [8], also underscores the fact that there is no neurodevelopmental and genetic predisposition as to how fast and easy fluent reading skills are acquired. A related point is that reading is not an innate ability, but a skill to be learned related to a cultural artifact, that is, the writing system. In this sense, learning to read is the same as learning to sing, dance or to play chess [4]. Some children excel in chess, while others are horrible even after years of practice, just like we see differences in children’s reading skills. So, what makes a reading disorder different from a chess disorder? In the following, we aim to examine dyslexia according to the essential characteristics of a disorder in general, and in specific, to those of a neurodevelopmental disorder.

Here, we argue in favor of the notion of dyslexia as a neurodevelopmental disorder by examining the harm and dysfunction associated with it. This view is not incompatible with the place of dyslexia at the tail of the continuum of reading abilities. However, importantly, it provides a necessary instrumental framework to plan informed interventions from which individuals with reading problems are the ultimate beneficiaries. We emphasize the insights from fundamental neuroscientific research as central to our theoretical understanding of complex abilities like reading, and, therefore, they should have an impact upon our characterization of reading disorders. Furthermore, we discuss the practical implications of this approach in the clinical and educational domains.

### Terminology

Before we can look into the evidence in favor or against dyslexia as a neurodevelopmental disorder, it is vital to start by clarifying the terminology. First, what constitutes a disorder? According to the Diagnostic and Statistical Manual of Mental Disorders (DSM-5), a mental disorder is a syndrome characterized by a clinically significant disturbance in an individual’s cognition, emotion regulation, or behavior that reflects a dysfunction in the psychological, biological, or developmental processes underlying mental functioning. Mental disorders are usually associated with significant distress or disability in social, occupational, or other important activities [9]. Thus, to be considered a disorder, the symptoms have to represent a dysfunction in the underlying neuropsychological processes, and they should cause harm to the individual due to a clinically significant disturbance in the individual’s functioning in society. That is, a condition needs to meet the requirements of both harm and dysfunction [10]. Furthermore, in DSM-5, neurodevelopmental disorders (which encompass specific learning disorders) are considered a group of conditions with onset in the developmental period, typically early in development, before the child enters school, and are characterized by developmental deficits that produce impairments of personal, social, academic, or occupational functioning [9,11]. More specifically related to the term neurodevelopmental disorder, it is assumed that the deficits represent a biologically-based deviation from the typical neurodevelopmental trajectory [12]. 

Finally, the term disorder is itself of dynamic nature. In particular, the concept of Research Domain Criteria (RDoC), and the network modeling approaches for understanding psychopathology are relevant vis-à-vis the interpretation of neurodevelopmental disorders. Instead of primarily focusing on symptoms for disorder classification, RDoC aims to provide a way to classify disorders based on multiple dimensions of observable behavioral and neurobiological measures [13,14]. Therefore, we will also discuss dyslexia within these conceptual frameworks.

## 2. Harm: Dyslexia and Its Impact on the Individual’s Quality of Life

Reading is an essential skill for functioning in our modern societies dominated by the written word. Reading is the basis for an individual’s ability to learn in an academic context, and is fundamental for academic success [15]. The workplace has become more and more literacy-dependent over the last decades, and even low-paid, insecure jobs require adequate literacy skills nowadays [16,17]. In addition to literacy’s role as an essential kernel to learning and employment opportunities, good literacy skills are needed to manage one’s health, stay socially active, make informed decisions and engage politically [18]. Literacy is, therefore, considered an essential life skill [19], and as such declared a fundamental human right by the United Nations Convention on the Rights of the Child [18,20]. Fluent reading is thus considered of primordial importance to obtain socio-economic success in our knowledge society.

The most persistent symptom of dyslexia is dysfluent reading. The failure to become a fluent reader is not simply a reflection of a developmental delay, but it is persistent throughout adulthood [21,22]. In fact, DSM-5 incorporates a reference to response to intervention (RTI) to include this resistance as a fundamental symptom for diagnosing dyslexia as a specific learning disorder. Indeed, studies on the effects of remedial teaching show that dyslexics’ reading fluency problems are highly resistant to specialized educational support and even after a prolonged period of school-based remediation, children with dyslexia typically remain dysfluent readers [23,24,25].

So it is obvious that the impairments in dyslexia limit one’s possibilities to fulfill his or her potential in life, as it interferes with a fundamental skill to function in our society. It has a negative impact on quality of life, and children with dyslexia are exposed to significant stressors and are at severe risk of negative psychosocial consequences, including symptoms of depression and anxiety [26,27]. Therefore, it is of primary interest to investigate to what extent dyslexia impacts quality of life, which ultimately defines its clinical relevance. A standard method to quantify the loss in quality of life due to a disease, handicap or disorder is the Quality-Adjusted Life Year (QALY) measure [28]. Using this measure, a study into quality of life of individuals with severe dyslexia (prevalence 4% of the population) in the Netherlands [29] revealed that they experience a substantial loss in quality of life, comparable to the loss of individuals diagnosed with epilepsy and with attention deficit hyperactivity disorder (ADHD). That study also highlights the importance of good identification and early intervention in terms of economic and societal costs. 

## 3. Dysfunction: Atypical Neurodevelopment of Reading Networks

Now that we have provided evidence for the harm related to dyslexia, we turn our attention to a more challenging element of disorder: dysfunction. One of the main arguments raised against dyslexia as a neurodevelopmental disorder refers to the fact that the core symptom, reading, is of a continuous nature, thus obstructing the ability to differentiate disordered from non-disordered presentations. This continuity problem is, however, not limited to dyslexia. Virtually every psychiatric symptom characterizing a mental disorder can occur in a certain gradation or form in a normally functioning person [30,31]. Moreover, in contrast to many medical conditions, in most cases the symptoms of mental disorders are much harder to be validated by measuring specific biomarkers (such as an objectively identifiable tumor) [32,33]. Consequently, establishing the presence of an internal dysfunction in mental disorders is much more challenging than that of harm. To do so, a disorder should be captured in a set of symptoms constructed in such a way that they cannot reasonably be considered normal, but lend support to an inference to underlying dysfunction or pathology [30]. To qualify symptoms in this context, some researchers use the term ‘pathosuggestive’ [30,31]. In this section, we address the state of research on neurobiological aspects related to atypical reading to provide a window on the element of dysfunction in dyslexia.

### 3.1. Brain Systems for Reading

Reading is a highly complex cognitive skill which is multimodal in nature. The act of reading must involve coordinated activity of both lower and higher-order processing brain areas, and can hardly be described by a single set of brain regions working independently. With this caveat in mind, neuroimaging research has been successful in delineating a set of brain systems that specialize for various sub-skills involved in reading, conforming a ‘reading network’. A failure in the specialization of one or several of those systems or in bringing them to act in concert is presumed to severely hinder fluent reading acquisition in dyslexics. 

Since the very early neuropsychological evidence [34], the advent of modern neuroimaging has considerably advanced our knowledge on the specific brain systems involved in reading. Studies on illiterate and late literate individuals have allowed us to directly investigate how learning to read transforms core brain function (see review in [35]). Such transformation has been hypothesized as a sort of ‘cultural recycling’ of brain areas which evolved for diverse basic functions and can be specialized to fulfil a culturally developed skill such as reading [36]. In addition, longitudinal research has provided insights into the developmental trajectory of specific neural activations with reading expertise (see reviews in [37,38]). Furthermore, the causal role in literacy of some of these brain systems has been supported by lesion studies examining patients with brain damage resulting in impaired reading (e.g., [39,40,41]). The neural basis for reading seems to combine fairly universal elements, such as specialization of visual areas to recognize a particular set of symbols, while other elements like the way symbols, sounds and meanings are integrated, seem to vary more from alphabetic to logographic languages [42]. So far, a primary contribution of the research reviewed above has been to neurocognitively decompose fluent reading into different sub-processes and the corresponding brain systems. Importantly, the various neuroimaging techniques allow for temporal and differential characterization of these components that may otherwise be elusive to behavioral tests. Thus, they reveal unique cognitive elements that are essential for a more thorough understanding of reading (dis)abilities.

### 3.2. Dysfunctional Brain Networks in Dyslexia

A growing number of studies has targeted the neurobiological basis of dyslexia and provided evidence in support for atypical development of brain networks in dyslexic readers. Most studies focused on specific brain regions previously associated with reading. With regard to neuroanatomy, several studies have suggested that dyslexics may present impaired structural connectivity across the main white matter tracts that constitute the anatomical basis of the reading network (see review in [43]), as well as asymmetries in key areas for auditory processing [44]. A larger corpus of studies has focused on functional neuroimaging to examine brain activity in a variety of reading-related tasks. Evidence from functional magnetic resonance (fMRI) studies shows differences in brain activations of specific brain systems [45,46]. Dyslexics seem to exhibit anomalous specialization of visual areas for word recognition [47], phonological processing in the auditory cortex [48,49], and integration of grapheme and phonemes in multisensory regions [50,51]. Several studies revealed that the very elemental neural process of recognizing whether a letter and a sound are matching or not, may remain deviant in adult dyslexics even after years of reading exposure, and thus being highly overlearned [52], further supporting the argument for atypical development. In addition, electroencephalography (EEG) and magnetoencephalography (MEG) studies compliment the fMRI findings with insights about their temporal specificity [53,54] and suggest additional deficits involving general brain oscillatory mechanisms [55,56,57,58]. More recently, the focus of part of neuroimaging dyslexia research has shifted towards connectivity, emphasizing the dynamic interplay between the systems in the reading network [45,59,60,61]. We should acknowledge that our understanding of neural dysfunction underlying dyslexia is currently still limited. A recent meta-analysis with focus on neuroanatomy highlighted inconsistencies across studies and the need for more large-scale research [62]. Notably, some of the inconsistencies may be explained by differences in selection criteria as many studies equate dyslexia to poor reading [63]. In any case, besides the incompleteness, not uncommon to neuroimaging research in general, qualitative and quantitative reviews strongly support a convergent pattern of abnormal brain activations underlying dyslexia across languages [45,64,65,66]. The terms dysfunctional and disrupted are often used in the literature when describing neural activations or networks in dyslexics vs. typical readers. This has been criticized by some authors on the basis that the findings merely indicate deviations in brain activity between two groups and no ‘core’ brain function is affected in dyslexia [4]. It has also been argued that a neurobiological origin does not necessarily imply brain dysfunction and a neurodevelopmental disorder, such as dyslexia, but may instead be considered as a natural product of ‘neurodiversity’ [67]. Although this argument seems reasoned, it is difficult to reconcile it with the need for dyslexia treatment in literate societies (harm). Thus, we argue that the denomination of dyslexia as a disorder and of the associated brain differences as dysfunctional are relevant. The brain findings in dyslexics reflect dysfunctionality as they demonstrate persistent deficits in enabling a skill with a high impact upon quality of life (see Section 2), and atypicality, as they show deviation from a reading network that is efficiently developed in the majority of individuals. Importantly, this does not necessarily imply a discontinuous distribution and categorical distinction between groups, similar to brain differences associated with other mental disorders such as autism, anxiety and depression.

### 3.3. Atypical Brain Development as Most Probable Cause

The notion that dyslexia is a neurodevelopmental disorder with a neurobiological component underscores that it is not caused by extraneous factors like socioeconomic status, poor instruction or lack of practice. The broad category of neurodevelopmental disorders include a heterogeneous group of conditions with a varying degree of genetic causes, which are in many cases poorly understood. The evaluation of genetic and environmental influences is important as it has consequences to clinical description, risk assessment and expected outcomes. In relation to this, as highlighted in a recent article, some of the assumed causal processes of dyslexia could in fact be also consequences of poor reading experience [68]. Dyslexics are likely to read less outside school settings and to have qualitatively poorer reading experiences and this fact complicates the distinction between cause and effect making it difficult to define underlying dysfunction [68,69]. In view of this, the explanatory value of the proposed neurobiological indicators of dyslexia may be compromised if they merely reflect the consequence of long-term poor reading instead of informing about an underlying cause. Importantly, however, several sources of evidence may be able to provide actual causal insights. First, some of the neural deviances in dyslexia are presumed to be present at birth or, at least, to precede reading onset [70,71,72,73,74,75,76,77]. Indeed, neural activity in pre-reading stages has been correlated with the probability of developing reading problems in later stages [72,74,78]. For example, Leppänen and colleagues revealed that infants neural responses to speech stimuli had a strong predictive power for reading fluency levels 14 years later [79]. In addition, differences in brain responses before reading onset have also been related to the risk dyslexia [71,80,81]. Moreover, there is evidence supporting the role of a genetic predisposition to the development of reading difficulties [82,83] and associated brain structural abnormalities [84,85]. Notably, a large behavioral–genetic twin study [86] showed that individual differences in reading ability are strongly heritable in contrast to those in reading exposure, and, importantly, that reading ability predicts reading exposure rather than vice versa. Altogether, these studies support the idea that dyslexia is unlikely to occur due to environmental factors. These findings suggest that in the context of dyslexia, less reading experience is not so much a cause, but is better seen as a mediating factor exaggerating symptoms in an atypically developing (reading) network. Interesting complements to this evidence are the findings from paradigms using artificial script learning in a controlled setting. Those studies show that neural sensitivity to newly learned orthographies [78] and performance in learning new script [87] in pre-readers can predict acquisition of reading skills in later years. Similarly, learning artificial letter-speech sounds predicted individual differences in reading and treatment outcomes in older children with dyslexia after a few years of reading instruction [88,89]. These results strengthen the causal role of atypical development rather than deficient teaching or poor reading experience. 

To sum up, there is compelling evidence suggesting that impairments in dyslexia may indeed be caused by atypical specialization of brain networks and begin early in development. This makes the case for dyslexia as a neurodevelopmental disorder according to the terminology of the DSM-5 and emphasizes the need for adequate identification and early specialized intervention.

## 4. Clinical and Educational Implications of a Neurodevelopmental Disorder Perspective

We believe that a neuroscience-informed view of dyslexia as neurodevelopmental disorder is useful and has positive clinical and educational outcomes that outweigh the alleged shortcomings (e.g., stigmatization, undermining the need for improvement in educational practices). 

A first benefit relates to the clinical specification of dyslexia. As we discussed in the previous section, symptoms differ in the extent to which they lend support to an inference to dysfunction, which is sometimes referred to as their pathosuggestiveness. One can argue that poor reading, being related to a learned skill, is in itself not particularly pathosuggestive, as it is a function of both quantity and quality of instruction, as well as potentially dependent on other external influences. However, the discussed evidence from neuroscientific studies, has yielded progress in characterizing dysfunction underlying reading deficits in dyslexia. A good example is the case of deficits in auditory-phonological processing and crossmodal letter-speech sound mapping, that typically co-occur with specific, persistent reading abilities, that are clearly more pathosuggestive. As discussed above, deficits in these fundamental neural processes precede and predict later reading failure, and are still deviant on the most basic level in adult dyslexics. Demonstrating co-occurrence of these deficits makes a strong case for an atypical or dysfunctional development of the neural reading network in children with dyslexia, thus anchoring it as a neurodevelopmental disorder. A behavioral validation of these biological markers comes from a study [90] showing that when dyslexia was defined on reading symptoms only, the percentages of affected pupils varied widely between schools. However, when the criteria for dyslexia included both reading and phonological–orthographic deficits, the percentages of positive cases hardly varied any more between schools [90]. This illustrates the utility of neuroscience to specify cognitive sub-skills that are important to reading fluency and improve our operational definition of dyslexia. In addition, studies with children at risk have shown improved prediction of reading difficulties when including neuroimaging measures over using behavioral tests only [91]. While the results still do not have a direct clinical application, neurobiological measures can help in disambiguating diagnostic criteria and refining current theories of reading disabilities [92]. For these purposes, an important success factor will be to find adequate markers of specific dysfunctions underlying dyslexia.

Furthermore, neuroimaging research can aid our definition and prediction of responsiveness to treatment. Currently, symptom persistency and resistance to remediation constitute an important factor in the diagnosis of dyslexia. The RTI framework encourages evidence-based instruction at different tiers of the school system and aims at better distinguishing children with poor reading due to external factors, like inadequate instruction, and those with a reading disability [93]. Nonetheless, there is no ample agreement on the inclusion of RTI in the definition of dyslexia [94,95], as this approach presents difficulties. It requires a definition of responsiveness that can be ambiguous and vary depending on the RTI model applied [96]. Perhaps more importantly, the operationalization of persistence in this approach can hinder early diagnosis of dyslexia, thereby delaying the application of specialized treatment [97], and prolonging the period of affliction in dyslexic readers. In addition, it conflicts with research showing that early interventions have a more favorable outcome than those provided at a later age [21,25,98]. Thus, we believe it is important to aim to include indexes of atypical neural development in diagnostic assessments in order to be able to detect dyslexia at earlier stages. To do so, one promising approach is to complement the RTI framework by a dynamic assessment approach, which focuses on factors moderating responsiveness and on the child’s learning potential [89,99]. As described above, there are promising efforts from neuroscience addressing differences between responders and poor responders [56,100,101] and simulating reading acquisition with artificial scripts [78,87] that could significantly contribute to our understanding of individual differences in treatment outcomes. 

### General Conclusions

In sum, unlike other learned abilities of similar complexity and specificity, e.g., playing chess, a persistent difficulty in learning how to read has a negative impact in an individual’s wellbeing, and thus is clearly harmful. Hence, we need to define a reading disorder but not a chess disorder. We believe there is compelling evidence suggesting that impairments in dyslexia may indeed be caused by atypical, specialization of brain networks, have a start early in development, and are persistent over time. This qualifies neurobiological mechanisms that impede a typical acquisition of reading skills as dysfunctional and makes the case for dyslexia as a neurodevelopmental disorder Additionally, we would like to emphasize that the view of dyslexia as a mental disorder is an essential tool to facilitate adequate support to individuals facing academic as well as psychological and/or socio-emotional problems. Although we acknowledge that the current model of disorders has conceptual limitations, we believe its utility is a matter of practical convenience for elaborating policies that can be effectively implemented in our healthcare systems. It is clear that not all poor readers have the same requirements when it comes to improving their reading. The more we understand about dysfunction in dyslexia, the more opportunities will arise to characterize individual differences and, thus, to develop tailored support. Eventually, this knowledge should facilitate a shift from the current, somewhat categorical, model of disorders to neuroscience-informed multidimensional models of individual differences. In this regard, the view of dyslexia as a neurodevelopmental disorder should also benefit from a more modern view of psychopathology, such as that derived from the network theory of mental disorders [102,103]. Whereas classification models as DSM-5 and the International Classification of Diseases (ICD-11) have a strong reliance on a classical, medical approach of disorder, in which an empirically identifiable condition unidirectionally causes a set of symptoms, these network theories aim to be more in line with the fact that in mental disorders complex, reciprocal influences between an atypical initial state (of the network), mediating factors and symptoms are present [102]. Basically, this theory conceptualizes disorders as a network of complex interactions between symptoms with direct causal connections between the symptoms that may be grounded on different levels (e.g., biological, psychological, societal, etc.). In the same vein, the RDoC approach of the National Institute of Mental Health (NIMH) promotes the conceptualization of mental disorders as dimensional rather than categorical, and the inclusion of different levels of analysis (e.g., neural circuit, neurocognitive, and behavior) that instantiate the constructs associated with mental disorders [104]. Multidimensional approaches also offer a framework to understand and investigate common factors among often co-morbid conditions, such as in the case of dyslexia and ADHD. These instrumental views, rather than that of a common underlying cause, offer an appealing organizational framework in which to accommodate the heterogeneity of behavioral and neuroscientific findings in the dyslexia literature and to advance the clinical valorization of insights.
